# Effects of Hot Water Extracts from* Polygonum multiflorum* on Ovariectomy Induced Osteopenia in Mice

**DOI:** 10.1155/2016/8970585

**Published:** 2016-09-26

**Authors:** Yun-Ho Hwang, Kyung-Yun Kang, Jong-Jin Kim, Sung-Ju Lee, Young-Jin Son, Soo-Heui Paik, Sung-Tae Yee

**Affiliations:** ^1^Department of Pharmacy, Sunchon National University, Suncheon, Republic of Korea; ^2^Singapore Bioimaging Consortium, Agency for Science, Technology and Research, 11 Biopolis Way, No. 02-02 Helios, Singapore 138667; ^3^Suncheon Research Center for Natural Medicines, Suncheon, Republic of Korea

## Abstract

*Polygonum multiflorum *(PM), a traditional Chinese medicine, is used to treat various diseases including nonalcoholic fatty liver disease and hyperlipidemia. However, the influence of PM on osteoporosis in animals is unclear. The present study investigated the antiosteoporotic effect of PM on bone mass in ovariectomized (OVX) mice and its possible mechanism of action. Twenty-five female C3H/HeN mice were divided into five groups of five mice as follows. Sham-operated control mice received daily oral gavage of an equal volume of water, and OVX mice received daily oral gavage of water or an injection of *β*-estradiol or PM for 6 weeks. Administration of PM significantly suppressed body weight and organs weight and increased weight and length of bone compared with the OVX group. Treatment with PM reversed osteopenia in OVX mice, thereby improving the bone morphometric parameters. Moreover, histological analysis using hematoxylin and eosin staining showed that PM inhibited OVX-induced bone loss. Serum estradiol and bone alkaline phosphatase levels were significantly decreased in the OVX group, with the levels increasing with PM treatment. In addition, tartrate-resistant acid phosphatase activity was inhibited by PM in OVX mice. These results suggest that PM is effective in preventing bone loss in OVX mice.

## 1. Introduction

Osteoporosis is a serious public health problem that occurs with aging populations [1, 2]. It is characterized by the loss of bone mass and microarchitectural deterioration, which leads to a high incidence of fragility fractures [3]. In the European Union, 22 million women and 5.5 million men currently have osteoporosis, with estimated 3.5 million new fragility fractures annually [4]. In many regions of the world, osteoporotic fractures may lead to the high therapeutic costs and mortality [5]. Osteoporosis is associated with inflammation and production of inflammatory cytokines including tumor necrosis factor-alpha (TNF-*α*), interleukin- (IL-) 6, and IL-1, which stimulate osteoclast activity [6]. Furthermore, nitric oxide (NO) produced upon activation of inducible NO synthase contributes to inflammation-induced osteoporosis by suppressing bone formation and causing osteoblast apoptosis [7]. Osteoporosis resulting from estrogen deficiency in menopausal females is most often caused by an increase in osteoclastic bone resorption compared with osteoblastic bone formation [8]. Thus, the treatment of osteoporosis focuses on agents that prevent bone loss or increase bone mass [9].

In females, estrogen has beneficial effects on the skeletal, cardiovascular, and central nervous systems [10]. Estrogen deficiency leads to increased bone loss through the activation of osteoclast differentiation. The deficiency can be prevented by estrogen replacement therapy (ERT) or hormone replacement therapy (HRT) [11]. Although many gaps exist in the understanding of the effects of postmenopausal HRT on health and illness, HRT is associated with increased risk of cardiovascular disease, breast cancer, and cholecystitis [12]. Therefore, it is necessary to develop alternative medicines with fewer side effects.

Traditional Chinese Medicine (TCM) has been used for thousands of years to treat various diseases, with many herbal remedies being effective in the improvement of female function. Some plant extracts contain estrogenic components, which have potential value in the treatment of menopausal symptoms [13].* Polygonum multiflorum *(PM, also known as Heshouwu in China) is one of the most popular TCM herbs used in the clinic for many diseases [14]. PM exhibits a variety of pharmacological efficacies that include neuroprotective effects [15], antioxidant activity [16], cytoprotective properties [17], and hair growth promotion [18]. 2,3,5,4′-Tetrahydroxystilbene-2-*β*-glucoside (TSG), an active polyphenolic component extracted from PM, displays protective effects on nephropathy in rats [19].

Estrogen deficiency in postmenopausal women is considered a state of the kidney. Kidney-nourishing Chinese herbs have been used for the treatment of bone loss resulting from estrogen deficiency [20]. Another study demonstrated that TSG protected MC3T3-E1 cells from hydrogen peroxide- (H_2_O_2_-) induced cell damage and inhibition of osteoblastic differentiation [21].

Emodin (3-methyl-1,6,8-trihydroxyanthraquinone) and physcion are two natural anthraquinone compounds [22]. Emodin reportedly has a variety of biological effects that include anticancer, hepatoprotective, antibacterial, anti-inflammatory, and immunosuppressive activities [23, 24]. Also, emodin regulates bone remodeling by inhibiting osteoclastogenesis and stimulating osteoblast formation [25]. TSG inhibits inflammatory responses by suppressing the expression of cyclooxygenase-2 (COX-2) and inducible nitric oxide synthase (iNOS) [26–28]. It has been suggested that TSG may suppress osteoporosis as immunological disorder [6].

There have been no studies investigating the protective effects of PM on ovariectomy- (OVX-) induced osteopenia in mice. We hypothesized that PM may beneficially prevent bone loss caused by estrogen deficiency. In this study, we show that PM hot water extracts inhibited bone deterioration in an OVX mouse model, suggesting a role of PM as a protective agent for mediating bone diseases.

## 2. Material and Methods

### 2.1. Chemical Reagents

TSG, emodin, chrysophanol, physcion, rhein, and dimethylsulfoxide (DMSO) and water soluble *β*-estradiol were purchased from Sigma-Aldrich Co. (St. Louis, MO, USA).

### 2.2. Preparation of the PM Extracts

PM was purchased from Dong-Bu Herbal Marker (Suncheon City, Republic of Korea). PM (500 g) was exhaustive maceration in water (3 × 5 L) at 85°C for 3 hours. The supernatant was filtered using Whatman number 2 filter paper before being transferred into preweighed containers. The fluid was concentrated with a rotary evaporator and freeze-dried to yield the crude extract (170 g).

### 2.3. Preparation of Standard Solution

TSG, emodin, chrysophanol, physcion, and rhein (1 mg) were dissolved in 1 mL of DMSO in a microtube. From the final solution, 200 *μ*L was transferred into a capped autosampler vial and 10 *μ*L was injected to a liquid chromatography-mass spectrometry (LC-MS) apparatus. The samples in autosampler were kept at 25°C during the experiment.

### 2.4. High-Performance Liquid Chromatography (HPLC)

HPLC was performed using a 1260-series apparatus, model 380 evaporative light scattering detector, and model 6130 quadrupole mass spectrometer (all from Agilent Technologies, Santa Clara, CA, USA) equipped with a Poroshell 120 SB-C18 column (150 × 4.6 mm i.d., 2.7 *μ*m particle size; Agilent Technologies;) with a compatible C18 guard column (4 × 3 mm i.d.; 3 *μ*m particle size; Phenomenex, Torrance, CA, USA). The mobile phase was composed of water (A; 0.1% formic acid) and acetonitrile (B; 0.1% formic acid) and was applied in a gradient of 0–2.00 minutes 95% A and 5% B, 2.00–23.00 min 100% B, and finally 23.0–27.00 100% B. The flow rate of the mobile phase was adjusted to 0.6 mL/min and the column temperature was set to 30°C. The injection volume was 10 *μ*L.

### 2.5. Optimization of HPLC and LC-MS Procedures

The mobile phase gradient of acetonitrile and water was satisfactory to determine the ionization abundance and separation of compounds. Quantitative MS enabled determination of ionization and collision energies. Quadrupole MS is commonly used due to its fragmented ion stability [29]. The optimum atmospheric-pressure chemical ionization-electrospray ionization (API-ES) parameters were determined as 12.0 L/min drying gas pressure, 3000 V capillary voltage positive and negative, 350°C drying gas temperature, and 35 psig nebulizer gas pressure. Mass spectrometric detector (MSD) positive and negative signal sets were 70 fragmentors, mass range 100–1000, and scan mode. Evaporative light scattering detector (ELSD) settings were gas flow rate 1.60 standard liter per min, 100% light emitting diode intensity, 30°C evaporate temperature, and 30°C nebulizer temperature.

### 2.6. Mice Osteoporosis Model

Eight-week-old female C3H/HeN mice (Orient Bio Inc., Iksan, Korea) weighing 20–22 g were used. Animals were housed in standard polycarbonate cages at 22 ± 2°C and 50–60% humidity on a 12-hour light/dark cycle with free access to commercial rodent chow (DAE-HAN Biolink, Daejeon, Korea). After acclimatization in the laboratory environment for one week, mice either were sham-operated (SHAM, *n* = 5) or received OVX (*n* = 20). In OVX animals, both ovaries were removed under Zoletil- and Rumpun-induced anesthesia. Animals were allowed to recover from surgery for 5 days prior to treatment. Mice were randomly divided into four groups of five animals each: (1) OVX mice receiving vehicle (water, oral); (2) OVX mice receiving *β*-estradiol water soluble (E_2_) at a subcutaneous dose of 0.03 *μ*g/head as a positive control; (3) OVX mice receiving PM at an oral dose of 100 mg/kg body weight (BW); (4) OVX mice receiving PM at an oral dose of 200 mg/kg BW. Water soluble *β*-estradiol was dissolved in distilled water to produce a stock solution of 10 mg/mL. The stock was diluted to 0.3 *μ*g/mL and was used for daily subcutaneous injections of 100 *μ*L (0.03 *μ*g) in the head. E_2_ and PM were administered for 6 weeks. BW was recorded weekly. At the end of the treatment, animals were sacrificed by cervical dislocation. Serum was collected and stored at −80°C until use. Spleen, thymus, uterus, and bone (tibia and femur) were removed and weighed. Femur and tibia length were measured using a Vernier caliper. All mice were treated in strict accordance with Sunchon National University Institutional Animal Care and Use Committee guidelines for the care and use of laboratory animals. All procedures were approved by the committee (approval number: SCNU IACUC-2015-01).

### 2.7. Serum Analyses

Before sacrifice, blood was acquired from the retro-orbital region of each ethyl ether anesthetized mouse. The serum was obtained by centrifugation at 5000 rpm for 5 min and was stored at −80°C until needed. Serum calcium (Ca), inorganic phosphorus (IP), alkaline phosphatase (ALP), and total cholesterol (TCHO) were determined using an automatic analyzer (Fuji Dri-Chem, Fuji, Japan) according to the manufacturer's instructions.

The amount of estradiol (E_2_) in the serum was analyzed by enzyme-linked immunoassay (ELISA) (Calbiotech, San Diego, CA, USA). Bone resorption status of mice in the different groups was assessed using a specific bone resorption marker (tartrate-resistant acid phosphatase (TRAP)). TRAP activity was also detected using ELISA (USCN Life Science, Wuhan, China). Bone formation was assessed by measurement of the bone formation marker bone specific alkaline phosphatase (BALP) using ELISA (Elabscience, Wuhan, China). All analyses were performed according to protocols provided by the manufacturers.

### 2.8. Microcomputed Tomography Analysis

Morphometric analysis was done to determine three-dimensional (3D) bone structure in vivo. We obtained bone morphometric parameters of the distal femora cleaned of adherent soft tissues including the bone volume/tissue volume (BV/TV), bone surface/tissue volume (BS/TV), bone surface/bone volume (BS/BV), trabecular thickness/separation/number (Tb.Th, Tb.Sp, and Tb.N), and volumetric bone mineral density (vBMD) after scanning with a SkyScan 1172 apparatus (SkyScan, Kontich, Belgium) and analyzing the volume of interest. Scans were taken with a source voltage of 49 kV and a source current of 200 *μ*A. Resolution was set to 17.09 *μ*m and the rotation step was 0.4°. Two-dimensional (2D) and 3D images were obtained for visualization and display. The structural parameters for trabecular bone were analyzed using the images and CTAn software (SkyScan).

### 2.9. Bone Histological Analysis

After extract administration, mice were sacrificed. Femur was removed and collected from each mouse. Each femur was fixed using 4% paraformaldehyde and processed for paraffin embedding. All samples were sliced at 7 *μ*m thickness and stained with hematoxylin and eosin (H&E) as well as Masson's trichrome stain. The sections were examined for changes in the trabecular bone and staining was visualized by microscopy. Images were captured using a camera.

### 2.10. Statistical Analysis

Differences in data between the groups are presented as the mean ± SD. Statistical differences were analyzed using Student's *t*-test. Probability (*p*) values < 0.05 were considered significant.

## 3. Results

### 3.1. LC-MS Analysis of PM

**Figure 1:**
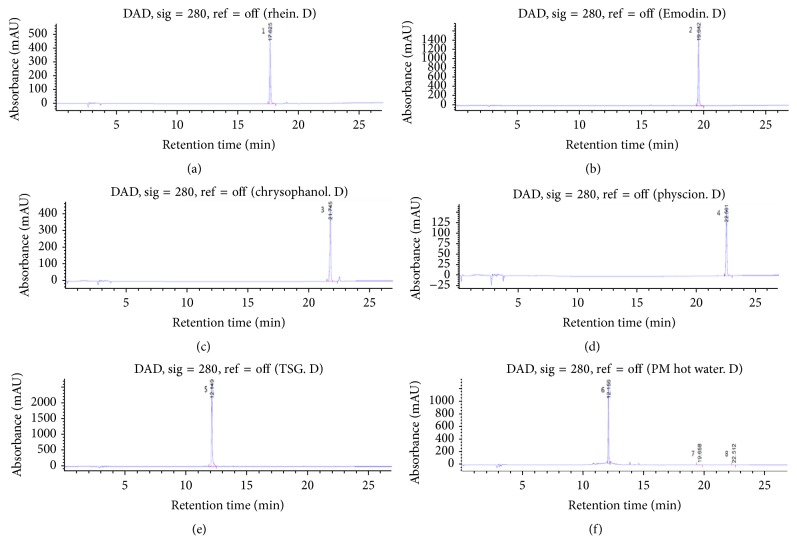
Chromatograms of* Polygonum multiflorum* (PM) and standard acquired at 280 nm. Chromatograms of the standards: (a) rhein, (b) emodin, (c) chrysophanol, (d) physcion, (e) 2,3,5,4′-tetrahydroxystilbene-2-O-*β*-D-glucoside (TSG), and (f) PM. Peak numbering is shown in Table 1.

LC-MS analysis of PM optimized the detection of the structures similar to those found to be more sensitive and which permitted the analysis of the target peak. Positive ion scan mode was employed; most of the constituents exhibited their quasi-molecular ions [M+H]+ and [M+Na]+. Combined LC-MS can provide online ultraviolet (UV) and MS information for each peak in a chromatogram. Generally, immediate identification of a peak is possible, based on a comparison with published data or with data derived from standard compounds. PM analyses have used five known standards; presently, the LC-MS and ELSD analyses of PM hot water extract used three standards: emodin, physcion, and TSG. Peak areas of emodin, physcion, and TSG were 0.9, 2.9, and 116.8 *μ*g/mg. Standard compounds were recorded as* m/z* values and compared with UV spectra. The analyses confirmed that PM harbored the three compounds. TSG mass in positive ion scan mode exhibited a peak at 407* m/z* for [M+H]+ and 429* m/z* for [M+Na]+ as the quasi-molecular ions. The respective peaks for emodin were 271* m/z* and 293* m/z* and were 285* m/z* and 307* m/z* for physcion as the quasi-molecular ions [30–32]. Detailed information on experiment parameters and chromatograms is given in Table 1 and Figure 1.

### 3.2. Effects of PM on BW and Uterus, Spleen, Thymus, and Bone Weight in OVX Mice

**Figure 2:**
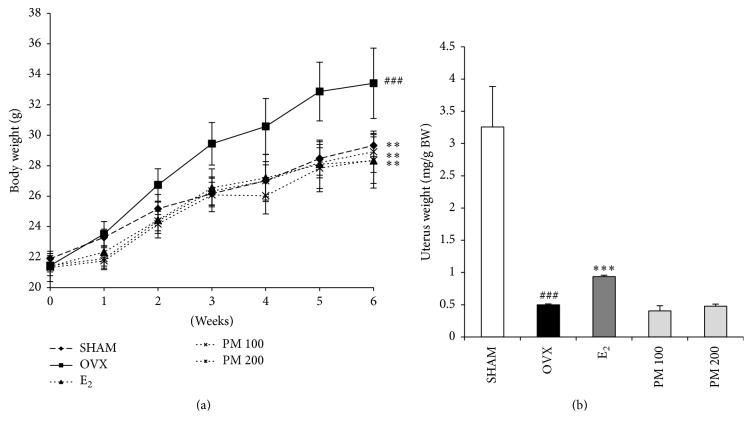
Effect on (a) body weight and (b) uterine weight after 6-week treatment. Each value represents the mean ± SD for *n* = 5. ^###^ *p* < 0.001 sham versus OVX group. ^*∗∗*^ *p* < 0.01 and ^*∗∗∗*^ *p* < 0.001, significantly different from ovariectomized mice.

As shown in Figure 2(a), the mice in all of the five experimental groups had similar initial body weights. Six weeks following the operation, the OVX mice showed a significant increase in final BW compared with the SHAM group (*p* < 0.001). Treatment with PM extract resulted in a significant reduction in OVX-induced weight gain in the OVX mice at both the 100 and 200 mg/kg doses (*p* < 0.01). Uterine weight of all OVX mice was significantly decreased compared with the SHAM group (*p* < 0.001), confirming the success of the surgical procedure, as the mice in the OVX groups experienced atrophy of uterine tissue. Uterine weight was not different between untreated OVX mice and OVX mice treated with 100 and 200 mg/kg PM extract (Figure 2(b)).

The effects of PM extract on thymus and spleen weights in OVX mice were assessed. Spleen and thymus weights were not different between the OVX and SHAM groups. However, the spleen weight in OVX mice treated with 100 and 200 mg/kg PM extract was significantly decreased as compared with the OVX group (*p* < 0.05). In addition, the thymus weight was decreased by treatment with both doses of PM extract (*p* < 0.001) (Table 2).

The effect of PM extract on bone weight and length was evaluated. Femur and tibia weights and lengths in the OVX control group were decreased. Supplementation with 100 and 200 mg/kg PM extract resulted in a significant increase in femur and tibia weight and length compared with the OVX group (Table 3).

### 3.3. Effects of PM on Bone Microarchitecture

**Figure 3:**
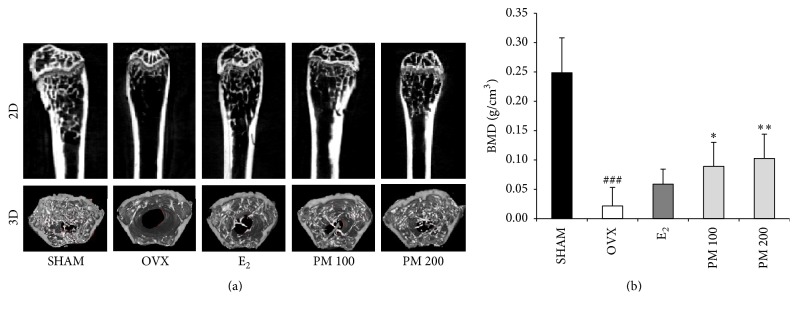
Effects of* Polygonum multiflorum* on ovariectomy induced deterioration of trabecular microarchitecture in femur. After the end of treatment, femurs were collected in 70% ethanol. (a) Representative two-dimensional (2D) images and three-dimensional (3D) images of the femur epiphysis. (b) Bone mineral density (BMD) was analyzed by *μ*CT after 6 weeks of PM administration. Each value represents the mean ± SD for *n* = 5. ^###^ *p* < 0.001 sham versus OVX group. ^*∗*^ *p* < 0.05 and ^*∗∗*^ *p* < 0.01, significantly different from ovariectomized mice.

**Figure 4:**
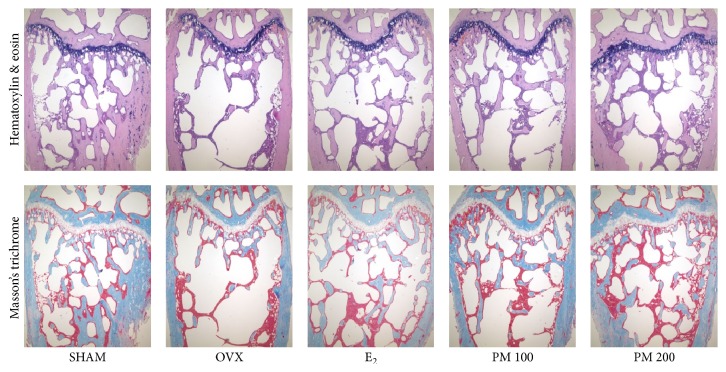
Histological analysis of distal femur with hematoxylin and eosin (H&E) and Masson's trichrome staining (×40 magnification).

**Figure 5:**
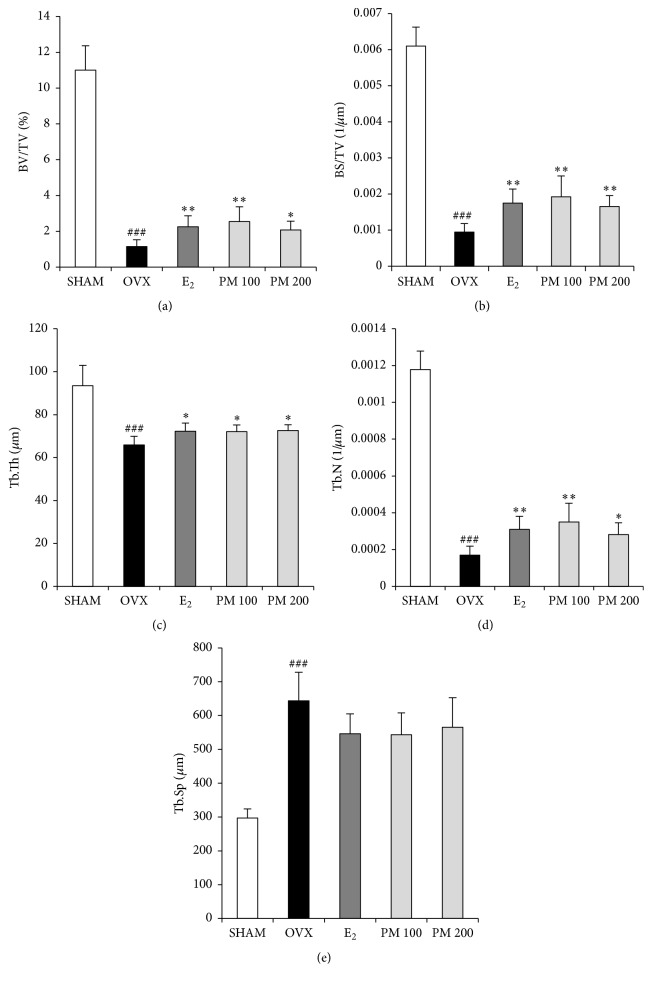
Effect of* Polygonum multiflorum* on trabecular morphometric parameters in distal femur of ovariectomized mice. Mice were treated with vehicle, PM (100, 200 mg/kg/day, p.o) for 6 weeks. (a) Bone volume/tissue volume (BV/TV), (b) bone surface/tissue volume (BS/TV), (c) trabecular thickness (Tb.Th), (d) trabecular number (Tb.N), and (e) trabecular separation (Tb.Sp) as analyzed with micro-CT SkyScan CTAn software. Each value represents the mean ± SD for *n* = 5. ^###^ *p* < 0.001 sham versus OVX group. ^*∗*^ *p* < 0.05 and ^*∗∗*^ *p* < 0.01 significantly different from ovariectomized mice.

To determine the effect of PM on OVX-induced deterioration of trabecular bone, bone mineral density (BMD) and bone microarchitecture were analyzed by micro-CT. The micro-CT images showed that oral administration of PM extracts at doses of 100 and 200 mg/kg to OVX mice prevented femoral bone loss (Figure 3(a)). BMD of the OVX group was decreased as compared with the SHAM group (*p* < 0.001); however, it was increased in both the 100 and 200 mg/kg PM-treated groups (*p* < 0.05 and *p* < 0.01, resp.) (Figure 3(b)). Changes in the trabecular bone of the femur were assessed by histological analysis. Compared with the SHAM mice, decreases in trabecular bone parameters were evident in the OVX mice. Treatment with PM protected against the deterioration (Figure 4). OVX altered the femoral trabecular architecture, but E_2_ and PM reduced the OVX-induced alteration (Figure 5). Compared with the SHAM group, the OVX group exhibited significant changes in bone volume density (BV/TV), bone surface density (BS/TV), trabecular thickness (Tb.Th), and trabecular number (Tb.N), suggesting that OVX caused significant loss of trabecular bone. PM extract treatment in OVX mice led to increased BV/TV and Tb.N at doses of 100 and 200 mg/kg (*p* < 0.01 and *p* < 0.05, resp.; Figures 5(a) and 5(d)), BS/TV at all doses (*p* < 0.01; Figure 5(b)), and Tb.Th at all doses (*p* < 0.05; Figure 5(c)). In contrast, trabecular separation (Tb.Sp) was increased compared with the SHAM group (*p* < 0.001), while treatment with PM extract did not cause any significant change (Figure 5(e)).

### 3.4. Effects of PM on Serum Biochemical Markers

Table 4 summarizes the results of serum biochemical parameters in animals in the different groups, following 6 weeks of oral administration of PM extract. As compared with the OVX group, the level of serum Ca was slightly lower in the PM-treated group but was statistically nonsignificant. The level of phosphorus in the OVX group was increased as compared with the SHAM group (*p* < 0.01) and was significantly decreased by treatment with PM extract at doses of 100 and 200 mg/kg (both *p* < 0.05). ALP was not different between the SHAM group and the OVX group. However, the ALP in OVX mice treated with PM extract at doses of 100 and 200 mg/kg was significantly decreased as compared with the OVX group (both *p* < 0.01). Serum TCHO was increased as compared with the SHAM group (*p* < 0.001), and treatment with PM at a dose of 100 mg/kg in OVX mice resulted in a significant reduction (*p* < 0.05).

### 3.5. Effects of PM on Serum TRAP, E_2_, and BALP

**Figure 6:**
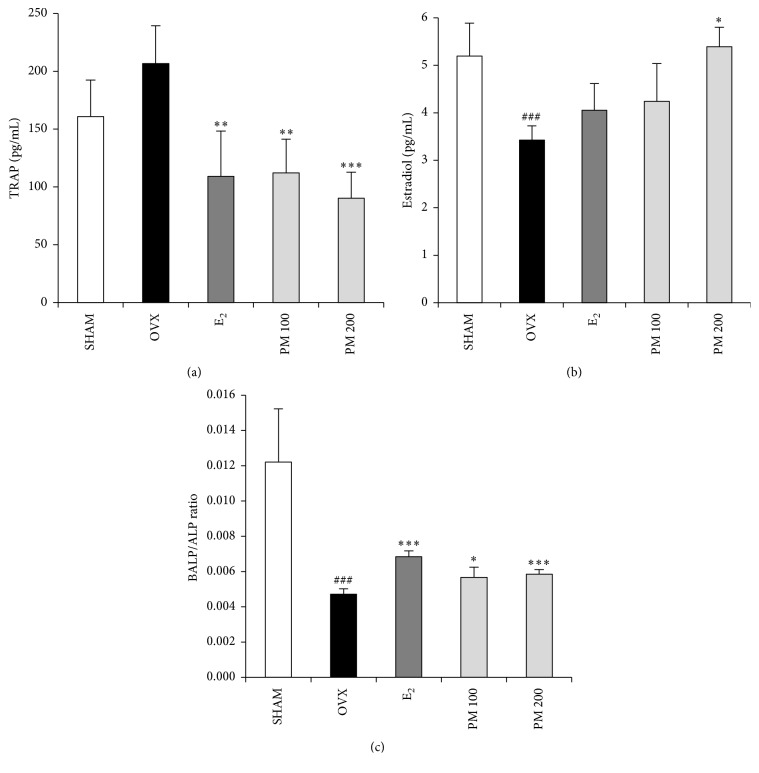
Effect of* Polygonum multiflorum* on serum tartrate-resistant acid phosphatase (TRAP), estradiol, and bone alkaline phosphatase/alkaline phosphatase (BALP/ALP). In control, SHAM-operated mice and OVX mice with or without the administration of PM (100, 200 mg/kg/day, p.o) for 6 weeks. Serum (a) TRAP, (b) estradiol, and (c) BALP/ALP ratios were measured by ELISA kit. Each value represents the mean ± SD for *n* = 5. ^###^ *p* < 0.001 sham versus OVX group. ^*∗*^ *p* < 0.05, ^*∗∗*^ *p* < 0.01, and ^*∗∗∗*^ *p* < 0.001 significantly different from ovariectomized mice.

To evaluate the effect of PM extract treatment on bone turnover in OVX mice, we measured the serum E_2_ and TRAP activity. As compared with the SHAM group, TRAP activity was slightly and statistically nonsignificantly increased in the OVX group. Treatment with 100 and 200 mg/kg PM in OVX mice resulted in a significantly decreased TRAP activity (*p* < 0.05 and *p* < 0.01, resp.; Figure 6(a)). The OVX group showed a significantly lower level of serum estradiol compared with the SHAM group (*p* < 0.001). The OVX group treated with 200 mg/kg PM had a significantly higher E_2_ level compared with the OVX group (*p* < 0.05) (Figure 6(b)). Furthermore, as compared with the OVX group, BALP as osteoblast activity marker was significantly increased in OVX group (*p* < 0.05 and *p* < 0.001) (Figure 6(c)).

## 4. Discussion

Hormone replacement therapy (HRT) is effective in the prevention of osteoporotic fractures and can improve the quality of life of women with postmenopausal symptoms. However, HRT reportedly increases risks of breast cancer, venous thromboembolism, heart disease, and stroke [33]. There is a need for an effective agent that prevents bone loss induced by estrogen deficiency with the least possible side effects.

The present study demonstrates the bone sparing effect of PM. In OVX-induced osteoporosis, deterioration in trabecular bone microarchitecture clearly led to bone loss in mice. Oral administration of PM effectively prevented trabecular bone loss and improved bone microstructure. Furthermore, TRAP activity, a marker of bone turnover, was decreased, and the E_2_ level was increased in PM-treated animals. These results suggest that PM protects against OVX-induced bone loss that is associated with the suppression of bone resorption.

Since estrogen deficiency directly affects the weight of the uterus, atrophy of the uterus is used as evidence of the success of OVX [34]. OVX decreases uterus weight and increases BW. These changes can be inhibited or reversed by E_2_. In the present study, OVX mice displayed increased BW and decreased uterus weight, confirming success of the operation. BW was decreased by PM treatment, but PM had no effect on uterus weight in OVX mice. OVX also induces increases in thymus and spleen weight, which contributes to increased thymic T and B cells, leading to more bone loss [35]. Presently, OVX mice treated with PM had reduced thymus and spleen weights. Another study reported that bone weight and length in OVX mice were lower than in sham-operated mice [36]. Significant changes in the weight and length of the tibia and femur were also detected in the present study. Treatment with PM significantly improved the weight and length of bone compared with the OVX group. These results suggest that PM may have an estrogen-like effect [37].

Osteoporosis is a bone disease caused by low BMD and microarchitectural deterioration, which leads to increased bone fragility and fracture. Thus, BMD is used as a method for the evaluation of individuals at risk of osteoporosis [38]. Presently, OVX significantly decreased the BMD of the femur when compared with the SHAM group. PM treatment prevented the decrease according to BMD values in OVX mice. Furthermore, the effects of PM on femoral microarchitecture were only investigated by micro-CT, demonstrating increased trabecular BV/TV, BS/TV, Tb.Th, and Tb.N and decreased Tb.Sp in mice treated with PM compared with the OVX mice. We clearly show the positive effects of PM on the structure and density of femur trabecular bone through 3D-reconstruction of images and 3D-microarchitectural analysis.

Bone turnover markers have been widely used as a tool to measure the effects of drugs on bone remodeling. Bone turnover is increased in OVX mice [39]. Presently, PM decreased the levels of Ca, IP, and ALP for 6 weeks, suggesting that PM prevents the OVX-induced increase in bone resorption in mice.

Bone homeostasis depends on bone formation and resorption. Excessive bone resorption by osteoclasts relative to osteoblastic bone formation enhances microarchitectural deterioration of bone mass. The TRAP osteoclast-specific marker is increased in OVX mice [40, 41]. Treatment with PM presently decreased TRAP activity indicating that PM may function by inhibiting osteoclasts. To evaluate bone formation BALP was used an osteoblast activity marker [42]. Treatment with PM increased BALP activity. Deficiency in the sex hormones usually leads to body fat accumulation and loss of bone mass [43, 44]. In our experiments, oral administration of PM significantly affected the serum E_2_ concentration. The level of E_2_ in serum (Figure 6(b)) was significantly high in both PM groups, more so than E_2_ alone. The PM dose of 200 mg/mL elevated E_2_ compared to the SHAM group. Although this dose of PM was not able to completely restore the osteoporotic phenotype, the trabecular bone parameters of BV/TV, BS/TV, Tb.Th, Tb.N, and Tb.Sp were significantly changed, as was BMD.

In this study, we confirmed that PM hot water extract contains TSG, emodin, and physcion. In particular, TSG has a protective effect on H_2_O_2_-induced damage to osteoblasts damage and inhibits expression of COX-2 and NO [21, 26–28]. Emodin suppresses osteoclastogenesis and stimulates osteoblast formation [25].

## 5. Conclusions

Daily oral administration of PM water extract contributes significantly to the prevention or treatment of the development of bone loss induced by OVX in mice. PM prevented the OVX-induced loss of bone mass and deterioration of trabecular microarchitecture, thereby maintaining the structural integrity and biochemical quality of the bone. Our results suggest that PM water extract may have therapeutic potential for the protection of postmenopausal osteopenia.

## Figures and Tables

**Table 1:** HPLC-ESI/MS data and identities of compounds associated with peaks detected in the HPLC chromatograms of samples of *Polygonum multiflorum*.

Peak^a^	Retention time (min)	Identification	[M+H]+ (*m*/*z*)	[M−H]+ (*m*/*z*)	[M+Na]+ (*m*/*z*)	Λ_max_ (nm)
1	17.625	Rhein	—	283	307	256, 430
2	19.642	Emodin	271	—	293	288, 438
3	21.745	Chrysophanol	255	—	277	256, 428
4	22.501	Physcion	285	—	307	286, 436
5	12.149	2,3,5,4′-Tetrahydroxystilbene-2-O-*β*-D-glucoside	407	—	429	214, 320
6	12.156	2,3,5,4′-Tetrahydroxystilbene-2-O-*β*-D-glucoside	407	—	429	214, 320
7	19.658	Emodin	271	—	293	288, 438
8	22.512	Physcion	285	—	307	286, 436

^a^Peak shown in the chromatograms presented in Figure 1.

**Table 2:** Effect of *Polygonum multiflorum* on thymus and splenic weights in ovariectomized mice.

	SHAM	OVX	E_2_	PM 100	PM 200
Thymus weight (mg)	22.9 ± 2.05	26.5 ± 5.24	23.2 ± 1.06	19.3 ± 2.67^*∗*^	20.8 ± 1.09^*∗*^
Spleen weight (mg)	84.2 ± 7.68	82.7 ± 2.96	75.3 ± 8.22	63.7 ± 3.9^*∗∗∗*^	70.5 ± 3.6^*∗∗∗*^

Each value represents the mean ± SD for *n* = 5. ^*∗*^ *p* < 0.05 and ^*∗∗∗*^ *p* < 0.001 significantly different from ovariectomized mice. PM 100 and 100 mg/mL; PM 200 and 200 mg/mL.

**Table 3:** Effect on *Polygonum multiflorum* on weight and length in femur of OVX mice.

	Weight (mg)		Length (mm)	
	Tibia	Femur	Tibia	Femur
SHAM	40.48 ± 0.74	50 ± 0.67	18.55 ± 0258	15.528 ± 0.28
OVX	37.94 ± 0.97^##^	46.2 ± 1.49^###^	18.088 ± 0.121^##^	15.015 ± 0.193^#^
E_2_	39.26 ± 0.95	48 ± 1.56	18.492 ± 0.128^*∗∗∗*^	15.834 ± 0.172^*∗∗∗*^
PM 100	39.22 ± 1.29	48.3 ± 1.43^*∗*^	18.508 ± 0.238^*∗∗*^	16.148 ± 0.202^*∗∗∗*^
PM 200	39.66 ± 0.8^*∗*^	48.1 ± 0.84^*∗*^	18.554 ± 0.215^*∗∗*^	15.834 ± 0.22^*∗∗∗*^

Each value represents the mean ± SD for *n* = 5.

^#^ *p* < 0.05, ^##^ *p* < 0.01, and ^###^ *p* < 0.001 sham versus OVX group.

^*∗*^ *p* < 0.05, ^*∗∗*^ *p* < 0.01, and ^*∗∗∗*^ *p* < 0.001 significantly different from ovariectomized mice.

**Table 4:** Effect of *Polygonum multiflorum* on serum biochemical markers.

	SHAM	OVX	E_2_	PM 100	PM 200
Ca (mg/dL)	10.22 ± 0.228	10.7 ± 0.158^##^	10.24 ± 0.207^*∗∗*^	10.48 ± 0.239	10.36 ± 0.404
IP (mg/dL)	3.84 ± 0.623	5.84 ± 0.979^##^	3.32 ± 0.716^*∗∗*^	4.28 ± 1.105^*∗*^	4.38 ± 0.669^*∗*^
ALP (U/L)	465.6 ± 43.707	439.6 ± 12.621	423.4 ± 14.977	404.6 ± 11.261^*∗∗*^	385.4 ± 32.936^*∗∗*^
TCHO (mg/dL)	128.4 ± 9.317	186.2 ± 9.23^###^	162.8 ± 6.611^*∗∗*^	174.6 ± 3.578^*∗*^	174.2 ± 17.254

Each value represents the mean ± SD for *n* = 5.

^##^ *p* < 0.01 and ^###^ *p* < 0.001 sham versus OVX group.

^*∗*^ *p* < 0.05 and ^*∗∗*^ *p* < 0.01 significantly different from ovariectomized mice.

PM 100 and 100 mg/mL; PM 200 and 200 mg/mL.
